# Similar Efficacy and Safety of Basaglar^®^ and Lantus^®^ in Patients with Type 2 Diabetes in Age Groups (< 65 Years, ≥ 65 Years): A Post Hoc Analysis from the ELEMENT-2 Study

**DOI:** 10.1007/s13300-018-0405-5

**Published:** 2018-03-14

**Authors:** Robyn K. Pollom, Timothy Costigan, Lyndon B. Lacaya, Liza L. Ilag, Priscilla A. Hollander

**Affiliations:** 1 Eli Lilly and Company, Indianapolis, IN USA; 2 Baylor Endocrine Center, Dallas, TX USA

**Keywords:** Age, Efficacy, Insulin, Safety, Type 2 diabetes

## Abstract

**Introduction:**

To compare efficacy and safety of Basaglar^®^ [insulin glargine 100 units/mL; LY insulin glargine (LY IGlar)] to Lantus^®^ [insulin glargine 100 units/mL; SA insulin glargine (SA IGlar)] in older (≥ 65 years) or younger (< 65 years) patients with type 2 diabetes (T2D).

**Methods:**

This subgroup analysis of a phase 3, randomized, double-blind, multinational, 24-week study compared LY IGlar and SA IGlar on several clinical efficacy (change in glycated hemoglobin (A1c), basal insulin dose, weight) and safety outcomes (incidence of adverse events, insulin antibodies, hypoglycemia incidence and rates) in patients either ≥ 65 or < 65 years.

**Results:**

Compared with patients aged < 65 years (*N *= 542), patients aged ≥ 65 years (*N *= 214) had a significantly longer duration of diabetes; lower baseline A1c and body weight; and body mass index; and were more likely to report prestudy SA IGlar use. Compared to patients < 65 years, patients ≥ 65 years needed a lower basal insulin dose and experienced lower body weight gain. There were no significant treatment-by-age interactions for the clinical efficacy and safety outcomes, indicating that there was no differential treatment effect (LY IGlar vs SA IGlar) for patients ≥ 65 years vs those < 65 years. Moreover, within each age subgroup, LY IGlar and SA IGlar were similar for all clinical efficacy and safety outcomes.

**Conclusions:**

LY IGlar and SA IGlar exhibit similar efficacy and safety in patients with T2D who are ≥ 65 years and in those < 65 years.

**Trial Registration:**

ClinicalTrials.gov trial registration: NCT01421459.

**Funding:**

Eli Lilly and Company and Boehringer-Ingelheim.

## Plain Language Summary

Plain language summary available for this article.

The aim of this phase 3 clinical study was to compare the efficacy and safety of two drugs, Basaglar^®^ (LY IGlar) and Lantus (SA IGlar), in patients with type 2 diabetes that were either 65 years of age and/or older or younger than 65 years of age. This study ran for 24 weeks. The factors used to measure efficacy were changes in glycated hemoglobin (A1c), insulin dose, and weight. The safety outcomes were incidence of adverse events, incidence and levels of insulin antibodies, and the incidence and rate of low blood sugar. Compared with patients less than 65 years of age (*N *= 542), patients 65 years of age and older (*N *= 214) had diabetes for a significantly longer time period; had a lower baseline A1c, body weight, and body mass index; and were more likely to report that they used SA IGlar prestudy. Compared to patients less than 65 years of age, patients equal to or older than 65 years of age showed significantly smaller increases in insulin dose and body weight. There were no significant treatment-by-age interactions for the efficacy and safety outcomes, indicating that there was no difference in treatment effect (LY IGlar vs SA IGlar) for patients equal to or older than 65 years of age vs those less than 65 years of age. Moreover, within each age subgroup, LY IGlar and SA IGlar were similar for all clinical efficacy and safety outcomes. LY IGlar and SA IGlar have similar efficacy and safety in patients with T2D who are equal to or older than 65 years of age and in those less than 65 years of age.

## Introduction

It is anticipated that from 2000 to 2030, the prevalence of diabetes among adults older than 64 years is expected to increase with estimates ranging from 48 million in developed countries to more than 82 million in developing countries [1]. Older adults with diabetes often present with other comorbid conditions that limit self-care abilities and impact health outcomes and quality of life [2]. Maintaining glycemic control can be challenging in this population because of cognitive deficits and increased functional decline, which may impact the ability to provide self-care [3]. Additionally, older adults are more likely to take multiple medications, which may contribute to increased risks, such as urinary incontinence, falls, and fractures [2]. Once diagnosed, many older adults with diabetes may remain under the care of a primary care provider who should customize treatment on the basis of the clinical and functional heterogeneity of this population [2, 4–6].

Insulin glargine is an initial insulin treatment option and part of basal-bolus therapy in patients with type 2 diabetes (T2D) who are not achieving glycemic control with their current treatment [7]. Older adults with T2D may benefit from insulin glargine treatment because of prolonged duration of action allowing for once-daily dosing and lower risk of hypoglycemia relative to neutral protamine Hagedorn (NPH) [8–10] or other comparators [10]. Basaglar^®^ [insulin glargine 100 units/mL, LY insulin glargine (LY IGlar); Eli Lilly and Company, Indianapolis, IN, USA] is the first authorized biosimilar insulin in the European Union [11]. LY IGlar has an identical primary amino acid sequence to that of Lantus^®^ [insulin glargine 100 units/mL, SA insulin glargine (SA IGlar); Sanofi-Aventis Deutschland GmbH, Frankfurt am Main, Germany] [11]. Both LY IGlar and SA IGlar have highly similar preclinical, efficacy, safety, and immunogenicity profiles in patients with type 1 diabetes and T2D [11–14]. To determine whether these similarities in efficacy and safety profiles of LY IGlar and SA IGlar are also true for older adults (≥ 65 years) with T2D, this subgroup analysis compared the efficacy and safety of LY IGlar to SA IGlar in patients with T2D from the ELEMENT-2 study on the basis of age (≥ or < 65 years) at study entry.

## Methods

### Study Design

The ELEMENT-2 study was a phase 3, multinational, randomized, double-blind, 24-week study in patients with T2D. Details of the ELEMENT-2 study have been previously reported [11], and a post hoc study from ELEMENT-2 is reported here. The study conduct conformed to the ethical principles described in the Declaration of Helsinki [15] and written informed consent was obtained from all patients. The ELEMENT-2 study was registered at ClinicalTrials.gov (NCT01421459).

Adult patients (≥ 18 years) with T2D were included in the study if they were either insulin-naïve or reported prior SA IGlar treatment, received at least two oral antihyperglycemic medications (OAMs) with or without SA IGlar during the 12 weeks before screening, had a body mass index (BMI) ≤ 45 kg/m^2^, and had glycated hemoglobin (A1c) levels ≥ 7.0% and ≤ 11.0% (if insulin-naïve) or ≤ 11.0% (patients with prior SA IGlar experience). Patients were excluded if they reported prestudy treatment with pramlintide or insulin other than SA IGlar within the previous 30 days, received basal-bolus therapy, required a total daily insulin dose of at least 1.5 U/kg, or experienced more than one severe hypoglycemic episode within the prior 6 months [11].

On the basis of their randomization allocation, patients who reported prestudy SA IGlar at study entry received an initial dose of LY IGlar or SA IGlar that was the same as their prestudy SA IGlar dose. Patients who were insulin-naïve at randomization received an initial 10 U/day dose of LY IGlar or SA IGlar [11]. During the 12-week titration period, all patients followed a patient-driven titration schedule where 1 unit of basal insulin per day was added until fasting plasma glucose (FPG) levels ≤ 5.6 mmol/L (100 mg/dL) were attained [16]. Covered vials and insulin syringes were used to administer the assigned study treatment in order to maintain blinding [11].

### Statistical Analysis

The efficacy and safety of LY IGlar and SA IGlar were evaluated in patients aged ≥ 65 years and in patients aged < 65 years. Analyses were based on the full analysis set, which included all randomized patients who received at least one dose of study drug [11]. For insulin antibody level assessment, the analysis population was defined as all randomized patients who received at least one dose of study drug and had a baseline and at least one post-baseline insulin antibody level assessment [14]. Prespecified analyses comparing LY IGlar to SA IGlar in both age subgroups included change from baseline in A1c at 24 weeks, the primary efficacy outcome, change in body weight, hypoglycemia (total, severe, and nocturnal), serious adverse events (SAEs), treatment-emergent adverse events, adverse events (AEs) leading to discontinuation, allergic events, injection site reactions, and insulin antibodies as a categorical outcome [treatment-emergent antibody response (TEAR)]. Post hoc analyses included the proportion of patients achieving A1c targets, basal insulin dose, FPG, insulin antibody levels and documented symptomatic hypoglycemia. Daily mean blood glucose was derived from the

7-point self-monitored blood glucose (SMBG) as an average across all the time points in the daily SMBG profile (i.e., pre-meal for each meal, post-meal of breakfast and lunch, bedtime, and 3 A.M.). SMBG profiles were collected three times in the 2 weeks before each clinic visit and measured using study-provided glucometers. TEAR was defined as having a percentage antibody binding of at least 1.26% for patients with nondetectable antibodies at baseline, or at least 1% (absolute) and 30% (relative) above baseline values for patients with detectable antibodies at baseline [14]. Hypoglycemia was defined as having a blood glucose ≤ 3.9 mmol/L (70 mg/dL), consistent with European Medicine Agency [17] and American Diabetes Association [18] guidelines.

An analysis of covariance (ANCOVA) model was used to analyze continuous data (A1c change, weight change); the ANCOVA model included baseline A1c, country, sulfonylurea use, time of basal insulin injection [A.M., (P.M. or bedtime)], and treatment as fixed effects, baseline value of response variable as a covariate, and subgroup (≥ 65 years, < 65 years) and subgroup-by-treatment interaction. The Mantel–Haenszel test was used to analyze categorical data. The Wilcoxon test was used to analyze treatment comparisons for insulin antibodies and hypoglycemia rate. SAS Version 9.1.3 (SAS Drug Development, Cary, NC, USA) was used to analyze data.

## Results

### Baseline Characteristics

Of the 756 patients enrolled, 214 (28.3%) were ≥ 65 years old and 542 (71.7%) were < 65 years old. Patients aged ≥ 65 years were more likely to be white, more likely to report prior SA IGlar use, have a significantly longer duration of diabetes, and have significantly lower A1c, body weight, and body mass index than patients < 65 years. Significantly fewer patients 65 years or older had normal renal function status. Other baseline characteristics were similar between both age subgroups (Table 1).

**Table 1:** Baseline demographics and patient characteristics

Variable	≥ 65 years (*N *= 214)	< 65 years (*N *= 542)	*p* value
Age, years	70.42 (4.35)	54.25 (7.77)	< 0.001
Age, LY IGlar/SA IGlar, years (%)	29.8/26.8	70.2/73.2	0.376
Sex, male, *n* (%)	103 (48.1)	275 (50.7)	0.572
Race, *n* (%)			< 0.001
American Indian or Alaska Native	2 (0.9)	36 (6.6)	
Asian	14 (6.5)	50 (9.2)	
Black or African American	9 (4.2)	49 (9.0)	
Multiple	0 (0.0)	3 (0.6)	
White	189 (88.3)	404 (74.5)	
Duration of diabetes, years	14.42 (7.42)	10.28 (6.17)	< 0.001
Weight (kg)	85.95 (18.60)	91.72 (19.80)	< 0.001
BMI (kg/m^2^)	30.70 (5.35)	32.37 (5.44)	< 0.001
Glycated hemoglobin (%)	8.06 (0.99)	8.43 (1.09)	< 0.001
Sulfonylurea use (yes), *n* (%)	183 (85.5)	447 (82.5)	0.332
Time of basal insulin injection [AM/(PM or bedtime)], %	47.2/52.8	50.6/49.4	0.420
Renal function status, *n* (%)			< 0.001
Normal GFR (> 90 mL/min/1.73 m^2^)	70 (32.7)	440 (81.2)	
Mild reduction in GFR (60–89 mL/min/1.73 m^2^)	112 (52.3)	88 (16.2)	
Moderate reduction in GFR (30–59 mL/min/1.73 m^2^)	31 (14.5)	13 (2.4)	
Basal insulin (%), SA IGlar/none	45.3/54.7	37.3/62.7	0.047

Data are mean (SD) unless otherwise indicated

*BMI* body mass index, *GFR* glomerular filtration rate, *LY IGlar* LY2963016 insulin glargine, *N* total number of patients, *SA IGlar* insulin glargine, *SD* standard deviation

### Efficacy

**Fig. 1:**
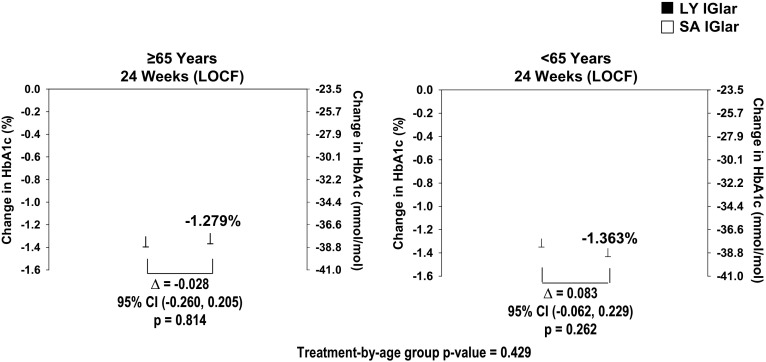
Baseline-to-endpoint changes in A1c in patients with type 2 diabetes ≥ 65 and < 65 years. Data are least squares mean ± standard error. CI confidence interval, LOCF last observation carried forward, LY IGlar LY2963016 insulin glargine, SA IGlar insulin glargine

Older (≥ 65 years) and younger (< 65 years) patients in both treatment groups showed similar reductions in A1c at the 24-week endpoint [last observation carried forward (LOCF), ≥ 65 years: least squares mean (LSM) ± standard error [SE] LY IGlar: − 5.6 ± 2.2%, SA IGlar − 5.6 ± 2.2%, *p* = 0.814; < 65 years: LY IGlar: − 5.6 ± 2.2%; SA IGlar: − 5.7 ± 2.2%, *p* = 0.262) (Fig. 1). No statistically significant age subgroup difference was observed for A1c (*p* = 0.700). The percentage of patients achieving their glycemic targets (A1c < 7%) was similar for both LY IGlar- and SA IGlar-treated patients in patients aged ≥ 65 years [LY IGlar: 55 (50.0%), SA IGlar: 55 (53.9%), *p* = 0.569] and in those aged < 65 years [LY IGlar: 125 (48.3%), SA IGlar: 142 (52.0%), *p* = 0.387].

**Fig. 2:**
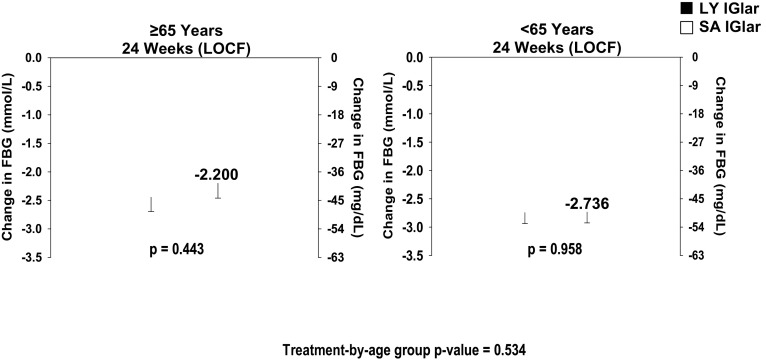
Baseline-to-endpoint changes in FBG by SMBG in patients with type 2 diabetes ≥ 65 and < 65 years. Data are least squares mean ± standard error. FBG fasting blood glucose, LOCF last observation carried forward, LY IGlar LY2963016 insulin glargine, SA IGlar insulin glargine, SMBG self-monitored blood glucose

Patients ≥ 65 years who were treated with LY IGlar or SA IGlar showed similar decreases in daily mean blood glucose (LSM ± SE, LY IGlar: − 2.074 ± 0.25 mmol/L, SA IGlar: − 1.919 ± 0.26 mmol/L, *p* = 0.617). Similar findings were observed in patients aged < 65 years (LY IGlar: − 2.250 ± 0.19 mmol/L, SA IGlar: − 2.382 ± 0.19 mmol/L, *p* = 0.487). There was no statistically significant effect of age for daily mean blood glucose (*p* = 0.086). No treatment differences between LY IGlar and SA IGlar were observed for FBG by SMBG in either age subgroup (Fig. 2).

**Fig. 3:**
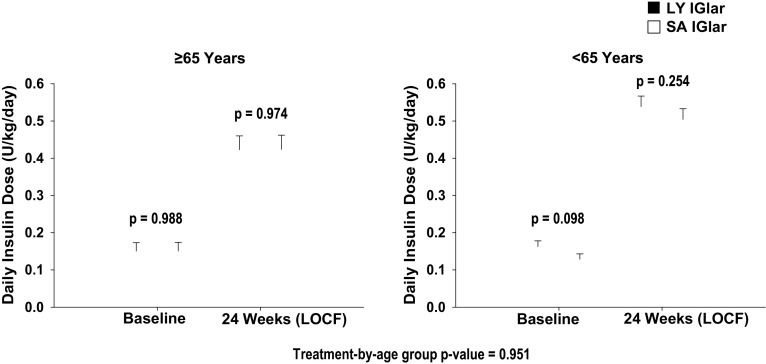
Baseline-to-endpoint changes in basal insulin dose in patients with type 2 diabetes ≥ 65 and < 65 years. Data are least squares mean ± standard error. FBG fasting blood glucose, LOCF last observation carried forward, LY IGlar LY2963016 insulin glargine, SA IGlar insulin glargine

Similar increases in basal insulin dose were observed in LY IGlar- and SA IGlar-treated patients across both age subgroups (Fig. 3). Basal insulin dose increased in both age subgroups at the 24-week endpoint (LOCF); however, the increase was significantly smaller in patients ≥ 65 years old (age group *p* < 0.001). Both treatment groups showed similar increases in body weight in patients ≥ 65 years old (LY IGlar: 1.412 ± 0.372 kg, SA IGlar: 1.397 ± 0.382 kg, *p* = 0.975) and < 65 years old (LY IGlar: 1.978 ± 0.281 kg, SA IGlar: 2.298 ± 0.279 kg, *p* = 0.282). However, patients aged ≥ 65 years exhibited statistically significantly smaller increases in body weight than patients under 65 years at the 24-week endpoint (LOCF) (age group *p* = 0.012).

### Safety

**Fig. 4:**
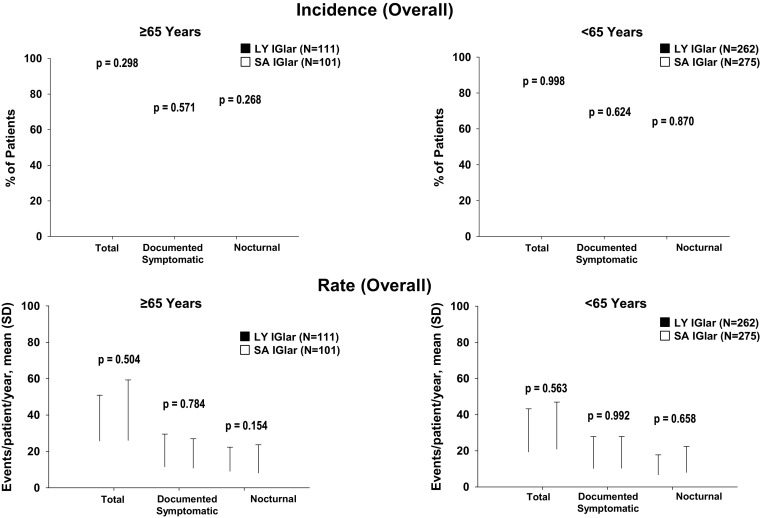
Overall incidence and rate of total, documented symptomatic, and nocturnal hypoglycemia in patients with type 2 diabetes ≥ 65 and < 65 years. Data for overall rate are presented as mean and SD. Hypoglycemia is defined as BG ≤ 3.9 mmol/L (70 mg/dL) or signs or symptoms of hypoglycemia. Overall refers to any time during the post-randomization visits. The treatment-by-age subgroup interaction values for the incidence of total, documented symptomatic, and nocturnal hypoglycemia are 0.362, 0.459, and 0.389, respectively. The treatment-by-age subgroup interaction values for rates of total, documented symptomatic, and nocturnal hypoglycemia are 0.737, 0.769, and 0.310, respectively. BG blood glucose, LY IGlar LY2963016 insulin glargine, SA IGlar insulin glargine, SD standard deviation

The incidence and 1-year adjusted rates of total, documented symptomatic, and nocturnal hypoglycemia were similar for both LY IGlar and SA IGlar, regardless of age subgroup (Fig. 4). Too few patients (≤ 10) experienced severe hypoglycemia for valid statistical analysis as prespecified in the Statistical Analysis Plan (LY IGlar: 3 patients; SA IGlar: 2 patients).

The overall proportion of patients with TEAR was similar in both treatment groups regardless of age subgroup [≥ 65 years, LY IGlar: 3 (2.8%), SA IGlar: 1 (1.0%), *p* = 0.363; < 65 years, LY IGlar: 11 (4.3%), SA IGlar: 13 (4.9%), *p* = 0.747]. Median insulin antibody levels (percent binding) were similar for LY IGlar- and SA IGlar-treated patients in both age subgroups (Table 2). Likewise, both treatment groups in each age subgroup showed similar incidences of AEs and SAEs (Table 3). Two patients (1 LY IGlar, 68 years and 1 SA IGlar, 67 years) died during the study. Neither death was considered by the investigator to be related to study drug.

**Table 2:** Insulin antibodies in patients

	≥ 65 years				*p* value	< 65 years				*p* value
	LY IGlar		SA IGlar			LY IGlar		SA IGlar		
	*N*	*n* (%)	*N*	*n* (%)		*N*	*N* (%)	*N*	*N* (%)	
Proportion of patients with detectable antibodies										
Baseline	107	5 (4.7)	97	1 (1.0)	0.215	258	15 (5.8)	268	12 (4.5)	0.556
24-week endpoint (LOCF)	107	3 (2.8)	97	3 (3.1)	> 0.999	258	27 (10.5)	268	19 (7.1)	0.217
Overall^a^	107	12 (11.2)	97	4 (4.1)	0.071	258	44 (17.1)	268	36 (13.4)	0.275

	*N*	Median (Q1, Q3)	*N*	Median (Q1, Q3)	*p* value	*N*	Median (Q1, Q3)	*N*	Median (Q1, Q3)	*p* value
Endpoint median insulin antibody levels										
Baseline	5	2.32 (0.44–2.97)	1	0.96 (0.96–0.96)	> 0.999	15	0.71 (0.46–1.54)	12	0.44 (0.34–0.78)	0.251
24-week endpoint (LOCF)	3	1.96 (1.11–5.66)	3	0.45 (0.26–4.38)	0.383	27	0.99 (0.38–5.14)	19	0.65 (0.36–2.76)	0.616

Values for *N* included in the analysis comprised only patients with detected or non-detected insulin antibody levels at baseline and post-baseline

The unit of measurement for insulin antibodies is percent binding

*IQR* interquartile range, *LOCF* last observation carried forward, *LY IGlar* LY2963016 insulin glargine, *Q1* 25th percentile, *Q3* 75th percentile, *SA IGlar* insulin glargine

^a^Overall refers to measurements taken during the 24-week treatment period and not at any specific visit or at endpoint (LOCF)

**Table 3:** Adverse events summary for patients ≥ 65 and < 65 years

Adverse events, *n* (%)	≥ 65 years		*p* value	< 65 years		*p* value	Treatment-by-age subgroup interaction
	LY IGlar *N *= 112	SA IGlar *N *= 102		LY IGlar *N *= 264	SA IGlar *N *= 278		
Patients with ≥ 1 TEAE	63 (56.3)	56 (54.9)	0.843	133 (50.4)	128 (46.0)	0.313	0.714
Special topic assessment^a^	5 (4.5)	7 (6.9)	0.447	16 (6.1)	20 (7.2)	0.597	0.695
Injection site reactions	4 (3.6)	1 (1.0)	0.211	9 (3.4)	8 (2.9)	0.723	0.337
Patients with ≥ 1 SAE	8 (7.1)	11 (10.8)	0.351	7 (2.7)	7 (2.5)	0.922	0.487

*INT* interaction, *LY IGlar* LY2963016 insulin glargine, *N* number of evaluable patients, *n* number of patients with TEAE, SAE serious adverse event, *SA IGlar* insulin glargine, *TEAE* treatment-emergent adverse event

^a^Categories of adverse events also include special topic assessment of adverse (allergic) events, injection site reactions, and SAEs though overall events are less than 5%

### Relationship Between Age and Clinical Outcomes

The change from baseline to endpoint (LOCF) for the clinical efficacy (Figs. 1–3 and *p* > 0.05 for weight) and safety (Fig. 4 and Tables 2 and 3) outcome measures was similar for each treatment group regardless of age. No statistically significant treatment-by-age interaction was observed for patients in either age subgroup.

## Discussion

The results of these subgroup analyses demonstrate similar clinical efficacy and safety outcomes within each age group for patients who receive LY IGlar or SA IGlar. Moreover, no effect of age was observed for any of the clinical efficacy and safety outcomes, except for basal insulin dose and body weight change. Older patients (≥ 65 years) required a lower basal insulin dose and gained less weight than younger patients (< 65 years). The effects of age on insulin dose and weight are consistent with previous reports of randomized controlled studies that evaluated insulin glargine in older (≥ 65 years) and younger (< 65 years) adults with T2D [9, 10].

This subgroup analysis of elderly patients (≥ 65 years) enrolled in the double-blind, phase 3 study showed similar hypoglycemic rates to patients under 65 years, which are consistent with hypoglycemia results seen in other studies comparing insulin glargine and NPH in older adults with T2D [9, 10]. In our subgroup analysis, 2 patients (1 LY IGlar, 1 SA IGlar) ≥ 65 years and 3 patients (2 LY IGlar, 1 SA IGlar) < 65 years reported severe hypoglycemic events.

The risk of hypoglycemia is an important consideration when treating older adults with T2D. Older adults may not recognize the signs of hypoglycemia, particularly if they have cognitive deficits or comorbid diseases that make self-monitoring of glucose challenging [5, 8, 19, 20]. In addition, with hypoglycemic events, there is an additional concern of related complications, such as injuries from falls [21]. Treatment guidelines recommend a glycemic-improving medicine with a lower risk of hypoglycemia for older patients at moderate risk of hypoglycemia [5]. Therefore, insulin glargine may be a useful treatment option in older patients because of its lower risk of hypoglycemia vs other comparators (e.g., NPH) [8–10, 19]. Combining insulin glargine with OAM, such as metformin or glimepiride, compared with premixed insulins has been effective in reducing A1c with a lower risk of hypoglycemia when OAMs are no longer effective in achieving glycemic targets [22].

In our subgroup analysis, AEs in the LY IGlar and SA IGlar groups were similar. Four LY IGlar patients ≥ 65 years reported injection site reactions (Table 3), which were characterized by rash or redness, or pain at the injection site, and were mild to moderate in severity, and the patients recovered from the event. One SA IGlar patient ≥ 65 years reported an injection site reaction, which was severe in intensity, but not characterized by rash or redness at the injection site and the patient recovered from the event.

Elderly patients with diabetes often have more comorbidities [2]; however, patients with significant cardiac disease and active cancers were excluded from our study. Therefore, the older age (≥ 65 years) subgroup including 34 (15.9%) patients (≥ 75 years) may have been more representative of an older population that has fewer comorbid health problems. Considering this study’s limitation, it is important to remember, as experts and professional organizations recommend, that health care providers need to customize treatment on the basis of a patient’s lifestyle, health status, risk factors, cognitive function, medical history, and social support [2, 4, 5].

## Conclusions

Our results demonstrate that LY IGlar and SA IGlar exhibit similar efficacy and safety in patients with T2D who are aged ≥ 65 years and in those who are aged < 65 years. For adult patients with T2D who require basal insulin as part of their treatment regimen, LY IGlar is an alternative basal insulin glargine that may be used with the same dose titration as SA IGlar.
